# Effects of subconjunctival administration of anti-high mobility group box 1 on dry eye in a mouse model of Sjӧgren’s syndrome

**DOI:** 10.1371/journal.pone.0183678

**Published:** 2017-08-24

**Authors:** Kyeong Hwan Kim, Dong Hyun Kim, Hyun Jeong Jeong, Jin Suk Ryu, Yu Jeong Kim, Joo Youn Oh, Mee Kum Kim, Won Ryang Wee

**Affiliations:** 1 Laboratory of Ocular Regenerative Medicine and Immunology, Seoul Artificial Eye Center, Seoul National University Hospital Biomedical Research Institute, Seoul, Korea; 2 Department of Ophthalmology, Haeundae Paik Hospital, Busan, Korea; 3 Department of Ophthalmology, Inje University College of Medicine, Busan, Korea; 4 Department of Ophthalmology, Gachon University Gil Medical Center, Incheon, Korea; 5 Department of Ophthalmology, Seoul National University College of Medicine, Seoul, Korea; Wayne State University, UNITED STATES

## Abstract

**Purpose:**

Extracellular high mobility group box 1 (HMGB1) acts as a damage associated molecular pattern molecule through the Toll-like receptor to promote autoreactive B cell activation, which may be involved in the pathogenesis of Sjӧgren’s syndrome. The aim of this study was to investigate the effect of subconjunctival administration of anti-HMGB1 on dry eye in a mouse model of Sjӧgren’s syndrome.

**Methods:**

Ten weeks-old NOD.B10.*H2*^*b*^ mice were subconjunctivally injected with 0.02 to 2 μg of anti-HMGB1 antibodies or PBS twice a week for two consecutive weeks. Tear volume and corneal staining scores were measured and compared between before- and after-treatment. Goblet cell density was counted in PAS stained forniceal conjunctiva and inflammatory foci score (>50 cells/focus) was measured in extraorbital glands. Flow cytometry was performed to evaluate the changes in BrdU^**+**^ cells, IL-17-, IL-10-, or IFNγ-secreting cells, functional B cells, and IL-22 secreting innate lymphoid cells (ILC3s) in cervical lymph nodes. The level of IL-22 in intraorbital glands was measured by ELISA.

**Results:**

Injection of 2 μg or 0.02 μg anti-HMGB1 attenuated corneal epithelial erosions and increased tear secretion (p<0.05). Goblet cell density was increased in 0.2 μg and 2 μg anti-HMGB1-treated-mice with marginal significance. The inflammatory foci score, and the number of BrdU^**+**^ cells, IL-17-, IL-10-, IFNγ-secreting cells, and functional B cells did not significantly change following anti-HMGB1 treatment. Surprisingly, the percentage of ILC3s was significantly increased in the draining lymph nodes (p<0.05), and the expression of IL-22 was significantly increased in the intraorbital glands (p<0.05) after administration of 2 μg anti-HMGB1.

**Conclusion:**

This study shows that subconjunctival administration of anti-HMGB1 attenuates clinical manifestations of dry eye. The improvement of dry eye may involve an increase of ILC3s, rather than modulation of B or plasma cells, as shown using a mouse model of Sjӧgren’s syndrome.

## Introduction

Sjӧgren’s syndrome represents one of the most devastating examples of autoimmune dry eye, which is involved in multiple pathological mechanisms and causes severe discomfort and visual disturbance. Many studies have shown the importance of type I interferon secreted by plasmacytoid dendritic cells, B cell responses, extracellular high-mobility group box 1 (HMGB1) and IL-17 pathways in Sjӧgren’s syndrome [1–3]. Current animal studies show that NOD.B10.*H2*^*b*^ mice are an excellent model of primary Sjӧgren’s syndrome, and B cells, plasma cells, or T helper 17 (Th17) cells are involved in the important pathogenic mechanisms in this mouse model [4–6].

**Fig 1:**
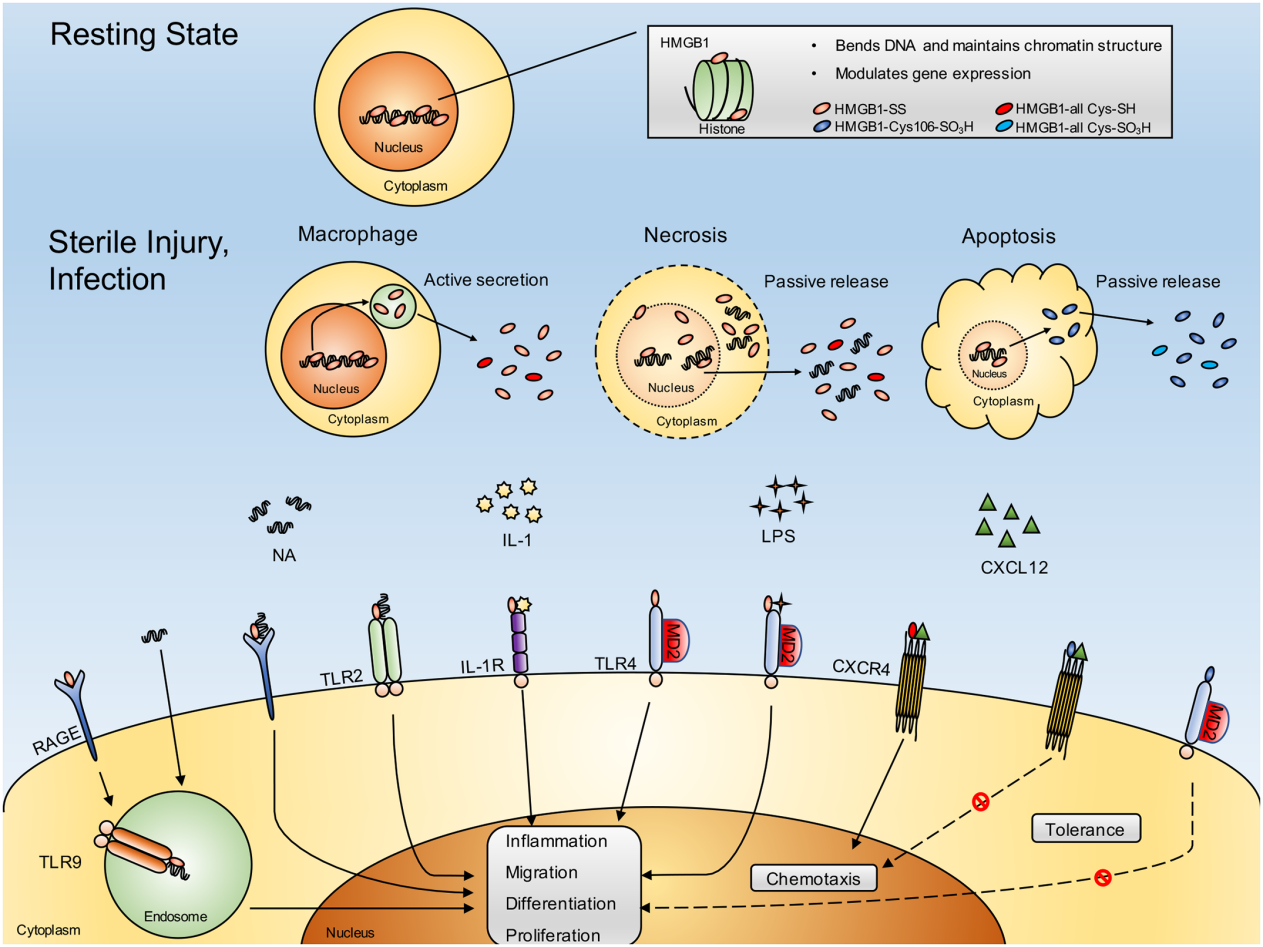
Intracellular and extracellular roles of HMGB1 protein. In the resting state, HMGB1 localizes to the nucleus, where it causes DNA bending and enhances the interaction of other proteins with DNA and their transcriptional activities. However, under conditions such as sterile injury or infection, HMGB1 is released either actively or passively into the extracellular space where it has distinctly different roles as a cytokine. The disulfide-bonded form of HMGB1, the usual form of extracellular HMGB1, elicits an inflammatory response, including dendritic cell maturation or autoreactive B cell activation, through specific receptors such as RAGE, TLR2, TLR4, or TLR9. Meanwhile, cysteine all-reduced HMGB1 does not have proinflammatory properties but behaves as a chemotactic cytokine through CXCR4. However, the cysteine all-oxidized form of HMGB1, which is produced during apoptotic cell death, loses the ability to induce inflammation and chemotaxis and gives rise to tolerance. HMGB1 = high mobility group box 1; HMGB1-SS = disulfide-bonded form of HMGB1; HMGB1-all Cys-SH = cysteine all-reduced HMGB1 (cysteines at positions 23, 45, and 106 of HMGB1 express a thiol group); HMGB1-Cys106-SO3H = cysteine 106-oxidized HMGB1 (cysteine at position 106 of HMGB1 expresses a sulfonic acid group); HMGB1-all Cys-SO3H = cysteine all-oxidized HMGB1 (cysteines at positions 23, 45, and 106 of HMGB1 express a sulfonic acid group); NA = nucleic acid; IL-1 = interleukin-1; LPS = lipopolysaccharide; CXCL12 = C-X-C motif chemokine ligand 12; RAGE = receptor for advanced glycation end products; TLR2 = toll-like receptor type 2; IL-1R = interleukin-1 receptor; TLR4 = toll-like receptor type 4; MD2 = lymphocyte antigen 96; CXCR4 = C-X-C motif chemokine receptor type 4; TLR9 = toll-like receptor type 9.

HMGB1 is a dual-function protein that has specific roles both inside and outside cells (Fig 1). HMGB1 is one of the most abundant non-histone nuclear proteins that contributes to chromatin stabilization, and contains two folded helical DNA-binding motifs, called A and B boxes. HMGB1 has three conserved redox-sensitive cysteines (C23, C45, C106), two located at positions 23 and 45 in the A box and one at position 106 in the B box. Modification of these cysteines determines the bioactivity of extracellular HMGB1 [7]. Extracellular HMGB1, which is passively released from necrotic cells or actively secreted by macrophages and dendritic cells, is a crucial cytokine that mediates the response to infection, injury, and inflammation, including autoimmune diseases such as Sjӧgren’s syndrome [2, 3, 8]. Necrosis- and pyroptosis-induced extracellular HMGB1 is usually in a disulfide-bonded form (between cysteine 23 and cysteine 45) that acts as a damage associated molecular pattern (DAMP) molecule through TLR 4, TLR 2, RAGE-TLR9, or IL-1R to promote dendritic cell maturation and autoreactive B cell activation. Extracellular HMGB1, in which all cysteines are reduced, binds to CXCL12 and acts through CXCR4 to cause cellular chemotaxis (Fig 1) [9–12]. In addition, extracellular HMGB1 is reported to be involved in the activation of Th17 cells during inflammatory disease [13], and may also be involved in IL-17 or IL-22 secretion in innate lymphoid cells (ILCs). ILCs are known to coordinate or limit immune responses during autoimmune disease, depending on environmental factors [14]. Conversely, apoptosis-induced extracellular HMGB1, in which all cysteines are oxidized, or cysteine 106 is oxidized, does not exhibit pro-inflammatory or chemotactic activities [7].

Innate lymphoid cells (ILCs) have emerged as a new type of immune cell with important functions in innate and adaptive immunity [14, 15]. Like natural killer (NK) cells, ILCs belong to the lymphoid lineage; however, unlike T and B cells, they lack antigen-receptors (T cell receptor or B cell receptor). ILCs are found in various tissues including the mucosa, lymphoid tissue, liver, skin, and fat. Group 1 ILCs consist of conventional NK cells and ILCs that secrete T helper (Th) 1-type cytokine IFNγ and express the transcription factor T-bet. Group 2 ILCs produce Th2-type cytokines IL-4, IL-5, or IL-13, and express the transcription factors ROR-α, Gata3, and T cell factor (TCF)-1. Group 3 ILCs include fetal lymphoid tissue-inducer (LTi) cells, and adult ILCs that either express or lack the natural cytotoxicity receptor (NCR, NKp46) (NCR^**+**^ILC3s and NCR^**-**^ILC3s, respectively). ILC3s produce the Th17-type cytokine, IL-17 or IL-22, and express the transcription factor ROR-γt. The function of ILCs in various tissues is described in Table 1, but it is not yet fully understood. In the intestine, ILC3s are known to promote epithelial wound healing and maintain epithelial barrier function.

**Table 1:** Functional characteristics of innate lymphoid cells (ILCs).

Type	ILC1	ILC2	ILC3
**Cells**	NK, ILC1	ILC2	LTi, NCR^**+**^ILC3, NCR^**-**^ILC3
**Transcription factor**	T-bet	ROR-α, Gata3, TCF-1	ROR-γt
**Tissue signal**	IL-12, IL-15, IL-18	IL-25, IL-33, TSLP	IL-1β, IL-23
**Effector cytokine**	IFNγ	IL-4, IL-5, IL-13	IL-17, IL-22, GM-CSF
**Function**	Macrophage activation Oxygen radicals	Mucus production Macrophage activation Tissue repair Vasodilation	Epithelial survival Anti-microbial peptide

NK: natural killer; LTi: lymphoid tissue inducer; NCR: natural cytotoxicity receptor (NKp46 in mice, NKp44 in human); T cell factor -1: (TCF)-1; TSLP: thymic stromal lymphopoietin

In Sjӧgren’s syndrome, the conjunctival, corneal, and lacrimal epithelial cells are damaged during inflammation, and some of them may undergo necrosis to release extracellular HMGB1. We hypothesize that extracellular HMGB1 in Sjӧgren’s syndrome may trigger strong auto-inflammatory cycles by activating an adaptive immune response and may establish a continuous pathological condition. Therefore, we investigated whether early treatment with an anti-HMGB1 blocking antibody could (1) improve the clinical manifestations of dry eye, (2) decrease the responses of autoreactive B cells or Th17 cells, and (3) affect changes in ILC3s that might be involved in modification of epithelial wound healing in NOD.B10.*H2*^*b*^ mice.

## Materials and methods

### Animals

All procedures used in this study strictly adhered to the ARVO statement regarding the Use of Animals in Ophthalmic and Vision Research. The experimental protocol was approved by the Ethics Committee at Seoul National University Hospital Biomedical Research Institute (IACUC No. 13–0162). Bilateral treatment was justified by the fact that this procedure was not visually disabling and that breeding of NOD.B10.*H2*^*b*^ mice was limited, according to ARVO Statement for the Use of Animals in Ophthalmic and Vision Research, and was approved by the Ethics Committee. NOD.B10.*H2*^*b*^ mice were purchased from Jackson Laboratories (Bar Harbor, ME, USA). All mice were bred in a specific pathogen-free environment and maintained at 22–24°C, relative humidity 55 ± 5%, with alternating 14/10 hour light/dark cycles (light-on 6 AM; light-off 8 PM) with free access to water and food at the mouse facility at the Biomedical Research Institute of Seoul National University Hospital. Overall health was monitored twice a week (weight and hair loss). Phenol red thread tests and corneal dye staining were performed following an intraperitoneal injection of zoletil (10 mg/kg) and xylazine (14 mg/kg), and all efforts were made to minimize suffering. Euthanasia was performed using compressed CO_2_ gas, according to the American Veterinary Medical Association Guidelines for the Euthanasia of Animals: 2013 Edition.

### Mouse model of Sjӧgren’s syndrome

Ten week-old male NOD. B10.*H2*^*b*^ mice were used as an autoimmune dry eye model, because NOD.B10.*H2*^*b*^ mice serve as a model for studying primary Sjӧgren’s syndrome without development of diabetes [5, 6, 16, 17]. To investigate inhibitory effect of triggering by HMGB1 in early stage of inflammatory cascades, 10 week-old mice were chosen based on previous preliminary data. A time course of clinical manifestations and inflammatory changes in the draining lymph nodes and intraorbital glands was evaluated between 10 and 16 weeks in BALB/C (n = 28), B6 (n = 32) and NOD.B10.*H2*^*b*^ mice (n = 34). Clinical dry eye and early inflammation was confirmed to have begun by 10 weeks (S1 Fig). For the current study, 24 male NOD. B10.*H2*^*b*^ mice were divided into treatment (n = 18) and control groups (n = 6).

### Anti-HMGB1 treatment

A chicken anti-HMGB1 polyclonal blocking antibody (product number: 326052233, SHINO-TEST Corporation, Kanagawa, Japan; 0.02, 0.2 and 2 μg/0.1 cc, n = 6 for each concentration) was injected subconjunctivally into both eyes twice a week for 2 weeks. A subconjunctival injection of the same volume of PBS containing 0.2 μg chicken Ig Y (Product Code: 326058471; SHINO-TEST Corporation, Kanagawa, Japan) served as a control. Three mice died during the experiments (two 0.02 μg anti-HMGB1-treated mice and one 0.2 μg anti-HMGB1-treated mouse) probably because of anesthetic overdose. Thereafter, we reduced the anesthetic dose to 20 μl.

### BrdU proliferation analysis

To evaluate proliferative changes in T and B cells, mice were given 0.8 mg/ml BrdU (BD Pharmingen^™^, San Diego, CA) in their drinking water for 10 days, and the water was changed every two days.

### Phenol red thread test for tear volume measurement

To evaluate tear production, phenol red-impregnated cotton threads (FCI Ophthalmics, Pembrooke, MA, USA) were applied to the lateral canthus for 60 seconds, and wetting of the thread was measured in millimeters. This was performed under anesthesia with zoletil (10 mg/kg) and xylazine (14 mg/kg) (n = 10–12). Twenty-four mice were included in the phenol red thread test for 10–11 weeks, and 21 mice were included in the test for 12 weeks and in the final analysis after sacrifice.

### Corneal dye staining

To evaluate the degree of corneal epithelial defects, one drop of 3% Lissamine Green B (Sigma-Aldrich, St. Louis, MO, USA) was administered to the inferior lateral conjunctival sac. This was convenient because there was no need for cobalt light excitation [18]. The corneal surface was observed (n = 10–12), and dye staining of the cornea was scored in a blinded assay as follows: a score of 0 indicated no punctuate staining; 1 indicated less than one third of the cornea was stained; 2 indicated two thirds or less was stained; and 3 indicated more than two thirds was stained. Twenty-four mice were included in the corneal dye staining test for 10–11 weeks, and 21 mice were included in the test for 12 weeks, and in the final analysis after sacrifice.

### Periodic acid schiff (PAS) staining for identification of goblet cells

The whole eyeball including the superior and inferior forniceal conjunctiva was excised and fixed in formalin. Tissues were cut into 4-μm-thick sections through the superior and inferior conjunctival fornices, and subjected to PAS staining. The total number of PAS-stained cells in the superior and inferior fornices of each eye was counted by two observers in a blind study (n = 16–24). Cell counts were averaged to determine goblet cell density in each group.

### Histopathology

The extraorbital glands were excised and fixed in formalin. Samples were cut into 4 μm sections and subjected to hematoxylin-eosin, CD3 (for T cells), or B220 staining (an isoform of CD45 and pan B cell maker). For CD3 and B220 immunohistochemical staining, rabbit anti-mouse CD3 (ab16669, Abcam, Cambridge, MA) and rat anti-mouse B220 (ab64100, Abcam, Cambridge, MA) primary antibodies were used. Anti-rabbit IgG HRP-linked antibody (#7074, Cell Signaling Technology, Danvers, MA) and goat anti-rat IgG H&L (HRP) (ab97057, Abcam, Cambridge, MA) were used as secondary antibodies for CD3 and B220 staining, respectively. The total number of inflammatory foci was counted in the CD3-stained slides (n = 7–9). A score of 1 was given when the focus contained greater than 50 CD3^+^ T lymphocytes [19].

### Flow cytometry

Draining cervical lymph nodes were minced between the frosted ends of two glass slides in RPMI media (WelGENE, Daegu, Korea) containing 10% FBS and 1% penicillin—streptomycin. Cell suspensions were collected and incubated for 30 minutes at 4°C with fluorescein-conjugated anti-mouse antibodies: CD3 (T cells), CD4 (T helper cells, monocytes and dendritic cells), CD8 (cytotoxic T cells, natural killer, and dendritic cells), CD19 (B cells and follicular dendritic cells), B220 (B cells), CD138 (plasma cells), Nkp46 (ILCs and natural killer cells), CD45 (all leukocytes). For IFNγ, IL-10, and IL-17A intracellular staining, cells were stimulated for 5 hours with 50 ng/ml phorbol myristate acetate and 1 μg/ml ionomycin in the presence of GolgiPlug (BD Pharmingen^™^, San Diego, CA). For IL-22 intracellular staining, cells were stimulated for 5 hours with 10 ng/ml IL-1β and 10 ng/ml IL-23 in the presence of GolgiStop (BD Pharmingen^™^, San Diego, CA). Non-specific staining was blocked using purified 2.4G2 Ab (BD Fc Block ^™^). Color conjugation combinations and gating strategies were as follows: (1) IFNγ, and IL-17A secreting cells; CD3-PerCP cy5.5 (eBioscience, 145-2C11), CD4-APC (eBioscience, GK1.5), CD8-PE cy7 (eBioscience, 53–6.7), B220-APC cy7 (eBioscience, RA3-6B2), IL-17A-PE (BD Pharmingen^™^, TC11-18H10), IFNγ-FITC (eBioscience, XMG1.2); (2) Plasma cells; CD3-PerCP cy5.5 (eBioscience, 145-2C11), B220-APC cy7 (eBioscience, RA3-6B2), CD138-APC (BD Pharmingen^™^, 281–2); (3) IL-10 secreting B cells; CD3-PE (eBioscience, 145-2C11), CD19-PerCP cy5.5 (eBioscience, eBio 1D3), B220-APC cy7 (eBioscience, RA3-6B2), IL-10-FITC (eBioscience, JES5-16E3); (4) ILC3s; CD3-PerCP cy5.5 (eBioscience, 145-2C11), B220-FITC (eBioscience, RA3-6B2), NKp46-APC (eBioscience, 29A1.4), CD45-APC cy7 (eBioscience, 30-F11), IL-22-PE (eBioscience, 1H8PWSR). Isotype controls were as follows: rat IgG2a,k for CD138-APC; rat IgG1,k for IL-17A-PE, IFNγ-FITC and IL-22-PE; rat IgG2b,k for IL-10-FITC.

Cells were assayed using a FACSCanto flow cytometer (BD BioSciences, Mountain View, CA). Data were analyzed using Flowjo software (Tree Star, Ashland, OR) (n = 4–6).

For BrdU (BD Pharmingen^™^, San Diego, CA) staining, a BrdU FITC cell cycle assay was performed (n = 4–6). During the acquisition preview, gates were adjusted in the FSC-A vs. SSC-A plot, and the DNA 7-AAD-A voltage was adjusted to place the mean of the singlet peak (G0/G1) at 50,000 on the histogram. In addition, cell cycle gates were adjusted as needed to encompass the G0/G1, S, and G2/M populations (S2 Fig).

To evaluate changes in ILC3s, cells were negatively gated with anti-CD3-PerCP and anti-B220-FITC antibodies, then positively gated with anti-CD45-APC cy7 and anti-IL-22–PE antibodies in the presence of anti-Nkp46-APC. Subpopulations of CD3^**-**^B220^**-**^CD45^**+**^Nkp46^**+**^IL-22^**hi**^ ILC3 cells (NCR^**+**^ ILC3s) and CD3^**-**^B220^**-**^CD45^**+**^Nkp46^**-**^IL-22^**hi**^ ILC3s cells (NCR^**-**^ ILC3s) were subsequently gated (S2 Fig). Fold changes in ILC3 percentage compared to controls were measured (n = 4–6).

### Enzyme-linked immunosorbent assay

Blood (800 μl– 1 ml) was obtained from the heart at the time of sacrifice. Plasma was collected after centrifugation at 2,500 rpm for 10 minutes, and used to measure the concentration of mouse anti-SSA (Ro-60, Signosis Inc. Santa Clara, CA) using an ELISA kit (Number EA-5202) according to the manufacturer’s protocol. Anti-SSA in the plasma was analyzed in PBS-treated mice (n = 6), in anti-HMGB1-treated mice (n = 12), and in the positive control provided with the kit (n = 2).

Intraorbital glands were minced into small pieces, and sonicated in PRO-PREP Protein Extraction Solution (Intron Biotechnology, Seongnam, Korea) on ice. The supernatant was collected after centrifugation at 12,000 rpm for 20 minutes, and assayed for IL-22 by ELISA, according to the manufacturer’s protocol (R&D Systems, Minneapolis, MN). Each sample was assayed twice (n = 8–12).

### Statistical analysis

GraphPad Software (GraphPad Prism, Inc., La Jolla, CA, USA) was used for statistical tests. To compare means from more than two groups, data were analyzed using the Kruskal-Wallis test. To compare means of two groups, data were analyzed using the Mann-Whitney test. To compare changes in ocular staining or tear secretion over time (baseline vs. post-treatment), which can be observed in vivo in living animals, data were analyzed using the Wilcoxon matched-pairs signed rank test. Data are presented as mean ± standard error (SE). Differences were considered significant at p < 0.05.

## Results

**Fig 2:**
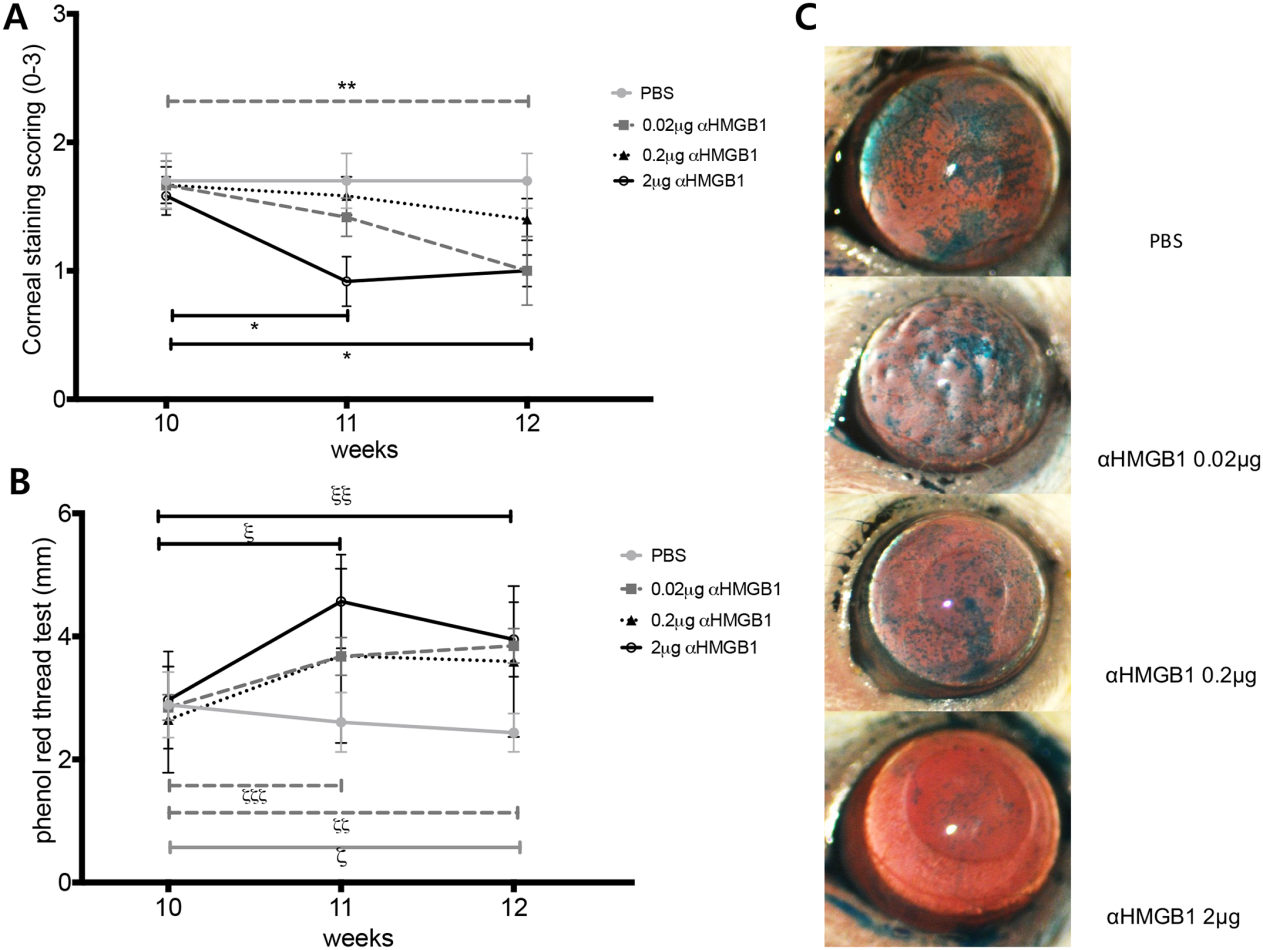
Ocular staining score and tear secretion in NOD.B10 mice after anti-HMGB1 treatment. (A) Decreased ocular staining score in 2 μg anti-HMGB1-treated mice (Wilcoxon matched-pairs signed rank test, 10 weeks vs. 11 weeks,* p = 0.0156; 10 weeks vs. 12 weeks, * p = 0.0156) and in 0.02 μg anti-HMGB1-treated mice (Wilcoxon matched-pairs signed rank test, 10 weeks vs. 12 weeks, ** p = 0.0313) compared to the baseline score. (B) Reduced tear secretion in PBS-treated control mice (Wilcoxon matched-pairs signed rank test, 10 weeks vs. 12 weeks, ^ς^p = 0.002). Increased tear secretion in 0.02 μg anti-HMGB1-treated mice (Wilcoxon matched-pairs signed rank test, 10 weeks vs. 11 weeks, ^ζ^p = 0.0273; 10 weeks vs. 12 weeks, ^ζζζ^p = 0.0078) and in 2 μg anti-HMGB1-treated mice (Wilcoxon matched-pairs signed rank test, 10 weeks vs. 11 weeks, ^ξ^p = 0.0005; 10 weeks vs. 12 weeks, ^ξξ^p = 0.0342) compared to the pre-treatment baseline. (C) Representative images of ocular staining scoring after treatment at 12 weeks. Data are presented as mean ± standard error.

**Fig 3:**
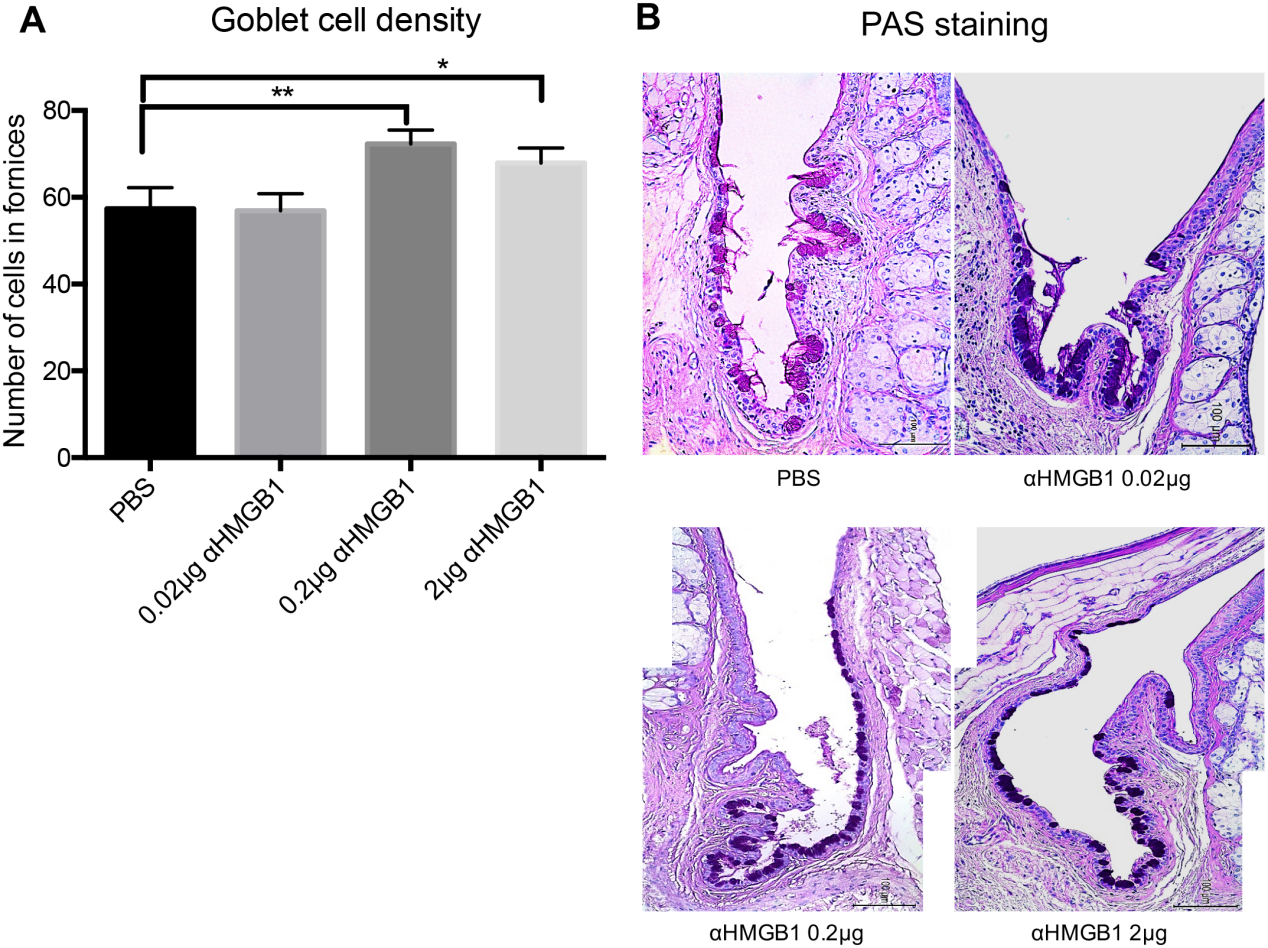
Goblet cell density in NOD.B10.*H2*^*b*^ mice conjunctiva after anti-HMGB1 treatment. (A) Increased goblet cell density in 0.2 μg anti-HMGB1- and 2 μg anti-HMGB1-treated mice (Mann-Whitney test, ** p = 0.0066, *p = 0.057; marginal significance). (B) Representative images of PAS staining of the forniceal conjunctiva (x200). Data are presented as mean ± standard error. Scale bar indicates 100 μm.

First, we looked at the effect of anti-HMGB1 treatment on the clinical manifestations of dry eye in NOD.B10.*H2*^*b*^ mice (Fig 2). The representative photos in panel C show the surface changes, and the ocular staining score was significantly decreased in 2 μg anti-HMGB1-treated mice over time, compared with the pre-treatment baseline level (Fig 2A and 2C; Wilcoxon matched-pairs signed rank test, 10 weeks vs. 11 weeks, p = 0.0156; 10 weeks vs. 12 weeks, p = 0.0156). The score was also decreased in 0.02 μg anti-HMGB1-treated mice compared to the pre-treatment baseline score (Wilcoxon matched-pairs signed rank test, 10 weeks vs. 12 weeks, p = 0.0313). The phenol red thread test (Fig 2B) showed that tear secretion was significantly reduced over time in PBS-treated control mice compared to the baseline (Wilcoxon matched-pairs signed rank test, 10 weeks vs. 12 weeks, p = 0.002). This was expected because of progression of the disease. On the other hand, tear secretion was significantly increased in 0.02 μg anti-HMGB1-treated mice (Wilcoxon matched-pairs signed rank test, 10 weeks vs. 11 weeks, p = 0.0273; 10 weeks vs. 12 weeks, p = 0.0078) and in 2 μg anti-HMGB1-treated mice (Wilcoxon matched-pairs signed rank test, 10 weeks vs. 11 weeks, p = 0.0005; 10 weeks vs. 12 weeks, p = 0.0342) compared to the pre-treatment baseline. In 0.2 μg anti-HMGB1-treated mice, ocular staining tended to be diminished and tear secretion tended to be increased, although neither were statistically significant. Goblet cell density was increased in 0.2 μg anti-HMGB1-treated (Mann-Whitney test, p = 0.0066 vs. PBS) and 2 μg anti-HMGB1-treated mice (Mann-Whitney test, p = 0.057 vs. PBS; marginal significance) 2 weeks after treatment (Fig 3).

**Fig 4:**
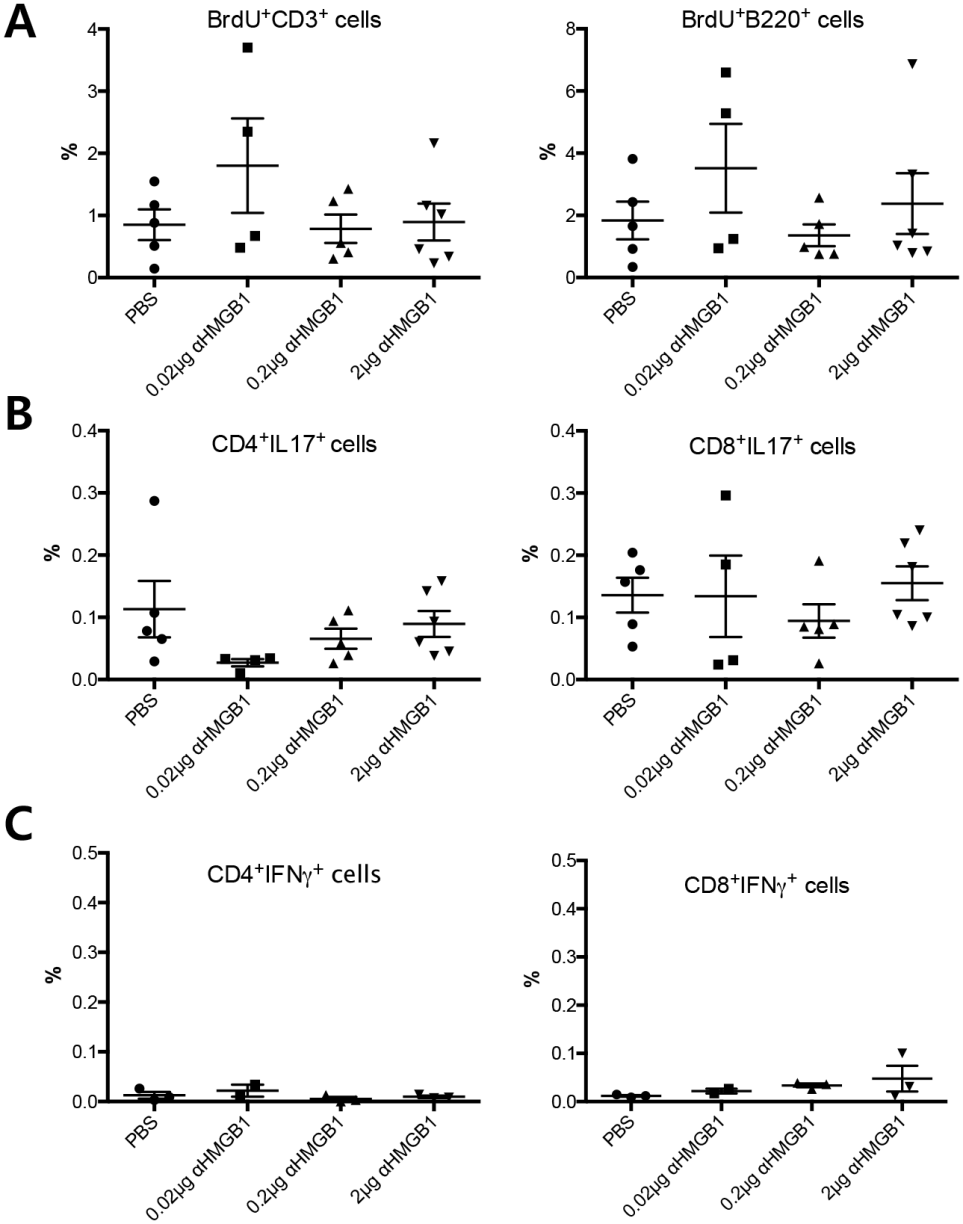
Changes in percentage of adaptive immune cells in draining lymph nodes of NOD.B10.*H2*^*b*^ mice after anti-HMGB1 treatment. (A) BrdU staining showing no significant proliferative changes in CD3^**+**^ T cells or B220^**+**^ B cells. (B) Percentage of Th17 (CD3^**+**^CD4^**+**^IL-17^**hi**^) cells and Tc17 (CD3^**+**^CD8^**+**^IL-17^**hi**^) cells were not altered after treatment. (C) IFNγ-secreting T cells (CD3^**+**^CD4^**+**^IFNγ^**hi**^ or CD3^**+**^CD8^**+**^IFNγ^**hi**^) were not affected by treatment. Data are presented as mean ± standard error. (Th17, T helper cells secreting IL-17; Tc17, cytotoxic T cells secreting IL-17).

**Fig 5:**
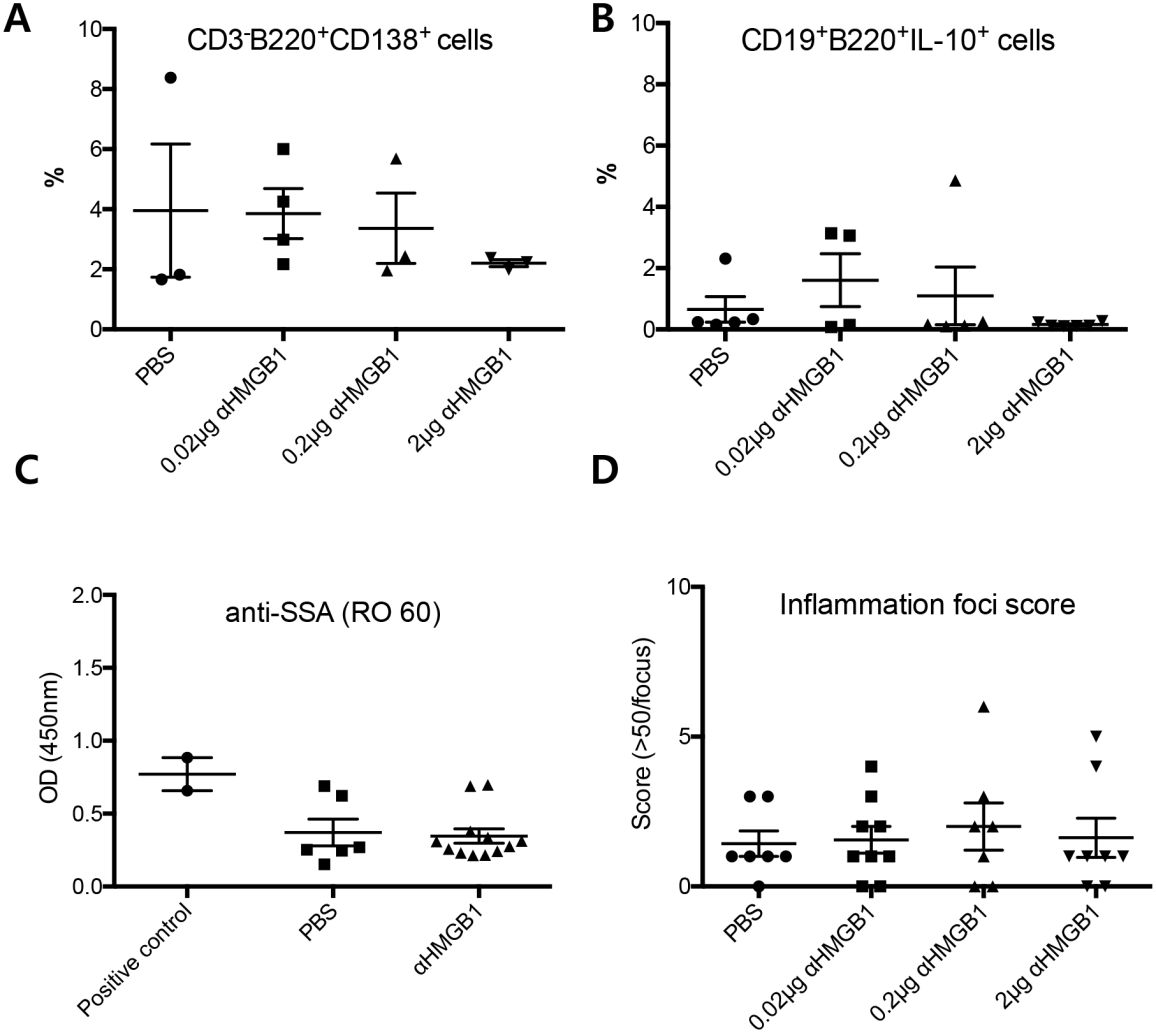
Changes in functional B cells in cervical lymph nodes, anti-SSA antibodies in serum, and inflammation foci scores in extra orbital lacrimal glands in NOD.B10.*H2*^*b*^ mice after anti-HMGB1 treatment. (A) No significant change in percentage of plasma cells (CD3^**-**^B220^**+**^CD138^**+**^ cells). (B) No change in IL-10-secreting B regulatory cells (CD3^**-**^CD19^**+**^B220^**+**^IL-10^**hi**^). (C) No change in the level of anti-SSA (RO 60) antibodies after treatment. (D) No significant changes in inflammatory foci scores (> 50 lymphocytes/focus) among all groups. Data are presented as mean ± standard error.

Next, we evaluated changes in immunologic responses in the extraorbital glands, plasma, and draining lymph nodes 2 weeks after treatment (Figs 4 and 5). The percentage and number of BrdU^**+**^CD3^**+**^ T and BrdU^**+**^B220^**+**^ B cells (S phase, S2 Fig) in the draining lymph nodes of treated mice were not significantly altered compared to that in controls (Fig 4A). For effector T cell responses, the percentage of Th17 cells (CD3^**+**^CD4^**+**^IL-17^**hi**^) and Tc17 cells (cytotoxic T cells that secrete IL-17; CD3^**+**^CD8^**+**^IL-17^**hi**^) did not show any significant changes (Fig 4B). IFNγ-secreting T cells (CD3^**+**^CD4^**+**^IFNγ^**hi**^ or CD3^**+**^CD8^**+**^IFNγ^**hi**^) were not changed following treatment (Fig 4C). For functional B cell responses, no significant changes were found in the percentage of plasma cells (CD3^**-**^B220^**+**^CD138^**+**^ cells) or IL-10-secreting B regulatory cells (CD3^**-**^CD19^**+**^B220^**+**^IL-10^**hi**^) in the draining lymph nodes (Fig 5A and 5B). Levels of anti-SSA were not significantly decreased in the plasma (Fig 5C). The infiltrating focus of CD3^**+**^ T cells was very similar to that of B220^**+**^ B cells (S3 Fig). Inflammatory foci scores in the extraorbital glands were not different among the groups (Fig 5D).

**Fig 6:**
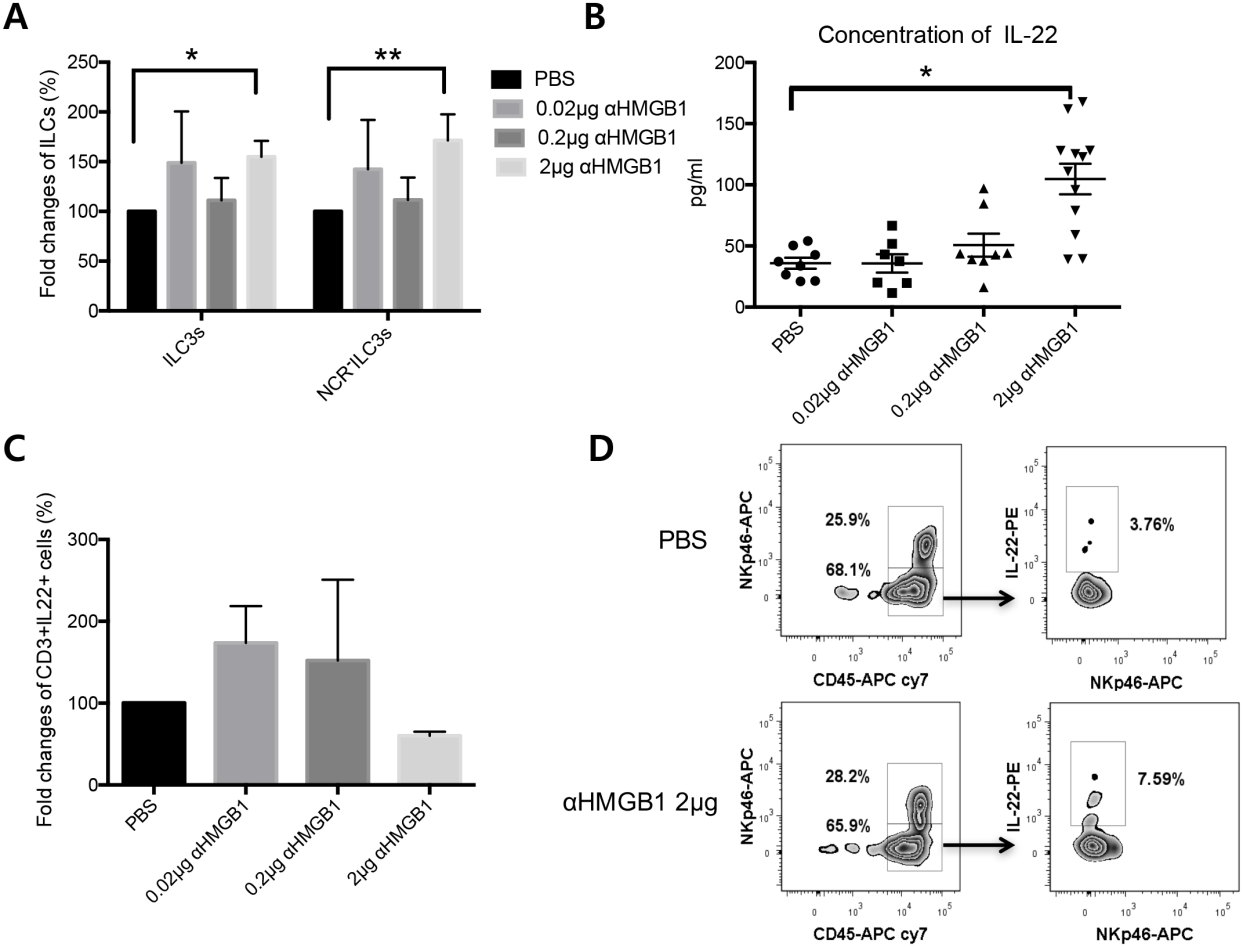
Changes in innate lymphoid cells (ILCs) in draining lymph nodes and changes in IL-22 expression in intraorbital glands after anti-HMGB1 treatment. (A) Fold changes in ILC3 percentage (CD3^**-**^B220^**-**^CD45^**+**^IL-22^**hi**^ cells; NCR^**+**^ or NCR^**-**^ ILC3) showing a significant increase following 2 μg anti-HMGB1 treatment compared with control (Kruskal-Wallis test, PBS vs. 2μg anti-HMGB1, *p = 0.025). Fold increase in NCR^**-**^ ILC3 percentage (CD3^**-**^B220^**-**^CD45^**+**^NKp46^**-**^IL-22^**hi**^ cells)(Kruskal-Wallis test, PBS vs. 2 μg anti-HMGB1, **p = 0.0142). (B) Increased IL-22 levels after 2 μg anti-HMGB1 treatment (Kruskal-Wallis test, PBS vs. 2 μg anti-HMGB1, *p = 0.025). (C) No change in percentage of CD3^**+**^IL-22^**hi**^ cells (Th22 cells or γδ T cells) in draining lymph nodes. Data are presented as mean ± standard error. (D) Representative images of NCR^**-**^ ILC3s (CD3^**-**^B220^**-**^CD45^**+**^NKp46^**-**^IL-22^**hi**^cells) in PBS- and 2 μg anti-HMGB1-treated groups. NCR, natural cytotoxicity receptor.

ILC3s are known to be involved in epithelial wound healing via secretion of IL-22. Therefore, we investigated changes in ILC3s in the draining lymph nodes 2 weeks after treatment (Fig 6A and 6D). Fold changes in the percentage of ILC3s (NCR^**-**^ILC3s; CD3^**-**^B220^**-**^CD45^**+**^Nkp46^**-**^IL-22^**hi**^ or NCR^**+**^ILC3s; CD3^**-**^B220^**-**^CD45^**+**^Nkp46^**+**^IL-22^**hi**^) were significantly increased compared to controls (Kruskal-Wallis test, PBS vs. 2 μg anti-HMGB1, p = 0.025). NCR^**-**^ILC3s (CD3^**-**^B220^**-**^CD45^**+**^Nkp46^**-**^IL-22^**hi**^) were also increased after 2 μg anti-HMGB1 treatment (Kruskal-Wallis test, PBS vs. 2 μg anti-HMGB1, p = 0.0142). IL-22 levels were significantly increased in the intraorbital glands after 2 μg anti-HMGB1 treatment (Kruskal-Wallis test, PBS vs. 2μg anti-HMGB1, p = 0.025, Fig 6B). There are three major populations of IL-22 secreting cells, namely Th22 cells, ILC3s, and γδ T cells. The percentage of CD3^**+**^IL-22^**hi**^ cells was not different among the groups, suggesting that the increase in IL-22 secretion is not related to Th22 or γδ T cells, which express CD3 (Fig 6C).

## Discussion

This study indicates that subconjunctival administration of anti-HMGB1 attenuates the clinical manifestations of dry eye. Furthermore, the data also suggest that the improvement in dry eye in NOD.B10.*H2*^*b*^ mice may involve an increase in IL-22-secreting ILC3s, rather than modulation of B or plasma cells.

The specific mechanisms that initiate inflammation in Sjӧgren’s syndrome are not fully understood, but we considered that the responses of reactive B cells and plasma cells that secrete autoantibodies could be key to maintaining the pathological condition [1]. Mouse models have clearly demonstrated the role of B and plasma cells in this process [4, 5]. We focused on extracellular HMGB1 because we believe that the chronic epithelial cell damage to the cornea or lacrimal glands may trigger the cycle of inflammation by secreting danger signals, such as HMGB1. It is plausible to assume that extracellular HMGB1 acts as an inflammatory cytokine through TLR9 signaling on B cells in this mouse model of Sjӧgren’s syndrome. Considering that TLR9 expression is abundant on B cells and plasmacytoid dendritic cells [20], we proposed that anti-HMGB1 treatment would reduce the effector function of B cells in these mice. However, B cell proliferation and the proportion of plasma cells, as well as anti-SSA levels, were not changed in the draining lymph nodes. We did not try to administer anti-HMGB1 systemically owing to the high cost. One reason for the failure to observe B cell modulation could be that the subconjunctival injection of anti-HMGB1 did not reach the spleen, where autoreactive B cells reside, at a sufficient therapeutic level to affect already activated B or plasma cells in the draining lymph nodes. In addition, B cell proliferation and the proportion of plasma cells were not affected following subconjunctival anti-HMGB1 treatment in the spleen (unpublished data).

It is also possible that early treatment with anti-HMGB1 did not have a measurable effect on inflammation because the epithelial damage was not sufficiently severe to secrete enough extracellular HMGB1 and induce inflammation. In addition, HMGB1 can increase Th17 cells by upregulating IL-6 or IL-23 [21–23]. Our study showed that anti-HMGB1 treatment did not affect the function of Th17 cells or Tc17 cells. Taken together, locally administered extracellular HMGB1 did not have a significant effect on adaptive immunity associated inflammation in this mouse model. We did not investigate changes in Treg cells after anti-HMGB1 treatment; therefore, further evaluation will be required to explore immune modulation following anti-HMGB1 treatment in more depth.

Despite the fact that we did not observe modulation of the effector function of B cells, the clinical manifestations of dry eye were still improved in this model. An increase in goblet cell density could indicate a protective effect due to an increase in tear secretion and a recovery of epithelial homeostasis. We therefore investigated the effect of IL-22-secreting ILC3 cells on epithelial damage in the dry eye model. This is based on the fact that ILC3 cells may play a role in epithelial homeostasis and wound healing as well as having a role in immune modulation [24–28].

ILCs are found mainly in mucosal tissues, including the intestinal tract (ILC3 and ILC2), lungs (ILC2), skin (ILC2), and tissues associated with lymphoid structures (ILC2) [29]. Emerging evidence shows that ILCs may function to maintain immune tolerance, homeostasis, or to inversely induce a T cell response by presenting foreign antigens or by secreting pro-inflammatory cytokines, depending on environmental cues and ILC subtype [14, 15, 28, 30, 31]. The circumstances that determine this bidirectional role of ILCs (immune regulation versus inflammation/autoimmunity) are still unclear. The protective function of ILC3 cells on epithelial homeostasis through IL-22 is well established in the gut, but their role in epithelial homeostasis in the eye has not been investigated. Under normal conditions, ILC3 distribution is scarce in the cervical lymph nodes (unpublished data), while inflammatory conditions, such as the mouse model for Sjӧgren’s syndrome (NOD.B10.*H2*^*b*^) or an experimental uveitis model, show a large number of ILC3 cells in the cervical lymph nodes (unpublished data). The increase in ILC3 cells in the draining lymph nodes and the increased IL-22 expression in the intraorbital glands after anti-HMGB1 treatment suggest a possible role for ILC3s in the improvement of dry eye in this model. Although we could not discriminate between changes in Th22 and changes in γδ T cells, the fact that the changes in CD3^**+**^IL-22^**hi**^ cells, including both Th22 and γδ T cells, were not significant suggests that the increase in IL-22 may be caused mainly by ILC3s. Our study showed a higher distribution of NCR^**-**^ILC3s (Nkp46^**-**^**)** and an increase in NCR^**-**^ILC3s (Nkp46^**-**^**)** in the cervical lymph nodes compared to NCR^**+**^ILC3s (Nkp46^**+**^**)** following treatment. These lineage-negative NCR^**-**^ ILC3s (CD3^**-**^B220^**-**^Nkp46^**-**^ CD45^**+**^IL-22^**hi**^ cells) are a subset of ILC3s known to effectively promote epithelial wound healing in the intestine [29, 32]. Our data suggest that NCR^**-**^ILC3s, rather than NCR+ILC3s, may function during dry eye.

Many cytokines show pleiotropic effects on cellular homeostasis depending on the concentration (lower or higher than normal endogenous levels). Extracellular HMGB1 also appears to have diverse effects on different cells, depending on the concentration or redox status of cysteine. Lower levels of endogenous HMGB1 may be required for optimal wound closure [33], while a higher level of HMGB1 acts as a pro-inflammatory cytokine and does not affect epithelial wound healing [12, 33]. HMGB1 with disulfide bonded-cysteine or reduced-cysteine exhibits inflammatory activities or chemotaxis, while HMGB1 with oxidized-cysteine exhibits tolerogenic activities [7, 34, 35]. In our autoimmune dry eye model, we believe that the level of extracellular HMGB1 is higher than endogenous levels, which then provokes inflammation. This could explain why blocking HMGB1 improves dry eye, although the exact redox status of cysteine is not known.

The present study had several limitations. First, the number of the animals was small owing to limited breeding. We were not able to directly reveal a plausible mechanism for how anti-HMGB1 treatment could cause an increase in ILC3. Given the fact that HMGB1 can interact with Treg cells or directly with conventional T cells and modulate them [36], there are several possible direct or indirect pathways through which HMGB1 could communicate with ILCs, which have not yet been discovered. This study was also unable to provide direct evidence of a correlation between the increase in ILC3s and clinical improvement. We did not perform adoptive transfer because of technical difficulties, since the number of ILCs was very small. Therefore, further studies to investigate the exact role of ILCs are warranted. Nevertheless, to our knowledge, this is the first study to report an improvement in dry eye after anti-HMGB1 treatment and to propose a possible role for ILC3s in this mouse model.

In conclusion, subconjunctival administration of anti-HMGB1 improved the clinical manifestations of dry eye in NOD.B10.*H2*^*b*^ mice, although the anti-HMGB1 treatment did not affect B or plasma cells.

## Supporting information

S1 Fig Preliminary experiments showing time course of clinical manifestation of dry eye and early inflammatory changes in cervical lymph nodes and intraorbital glands in NOD.B10.*H2*^*b*^ mice. Clinical manifestation of dry eye and inflammatory responses were evident by 10 weeks. (A) Significantly decreased ocular staining score in NOD.B10.*H2*^*b*^ mice at 10 weeks compared to BALB/c mice (Kruskal-Wallis test, **p < 0.01, ****p < 0.0001). (B) Phenol red thread test showing a significant decrease in tear secretion in NOD.B10.*H2*^*b*^ mice at 10 weeks compared to BALB/c mice (Kruskal-Wallis test, **p < 0.01, ***p < 0.001, ****p < 0.0001). (C) Goblet cell density in conjunctiva of NOD.B10.*H2*^*b*^ mice showing a decrease at 16 weeks compared to BALB/c mice (Kruskal-Wallis test, * p < 0.05). (D) Increased cell numbers in cervical lymph nodes of NOD.B10.*H2*^*b*^ mice at 10 to 14 weeks compared to 10 week-old B6 mice (Kruskal-Wallis test, * p < 0.05, **p < 0.01). (E-F) Increased IL-6 and BAFF levels in NOD.B10.*H2*^*b*^ mice at 10–12 weeks compared to controls (one-way ANOVA, *p < 0.05, **p < 0.01). RQ indicates a ratio of mRNA levels relative to controls. NOD, NOD.B10.*H2*^*b*^ mice.
(TIF)

S2 Fig Sorting BrdU^+^ cells and ILC3s. (A) ILC3s were negatively gated for anti-CD3 and anti-B220 antibodies and positively gated for anti-CD45 and anti-IL-22 antibodies. Subpopulation of NCR^**+**^ ILC3s (CD3^**-**^B220^**-**^CD45^**+**^Nkp46^**+**^IL-22^**hi**^ cells) and NCR^**-**^ ILC3s (CD3^**-**^B220^**-**^CD45^**+**^Nkp46^**-**^IL-22^**hi**^ cells) were subsequently gated. (B) During the acquisition preview, gates were adjusted in the FSC-A vs. SSC-A plot, and the DNA 7-AAD-A voltage was adjusted to place the mean of the singlet peak (G0/G1) at 50,000 on the histogram. In addition, cell cycle gates were adjusted as needed to encompass the G0/G1, S, and G2/M populations. NCR, natural cytotoxicity receptor.
(TIF)

S3 Fig Immunohistochemistry of the extraorbital glands of NOD.B10.*H2*^*b*^ mice. The infiltrating focus of CD3^**+**^ T cells (red arrows, upper panel, x400) almost matched that of B220^**+**^ B cells (red dashed arrows, lower panel, x400).
(TIF)
