# Supplementary figures and images for: Effects of subconjunctival administration of anti-high mobility group box 1 on dry eye in a mouse model of Sjӧgren’s syndrome
Source: PLoS One. 2017 Aug 24;12(8):e0183678. doi: 10.1371/journal.pone.0183678 (PMC5570279; doi:10.1371/journal.pone.0183678)

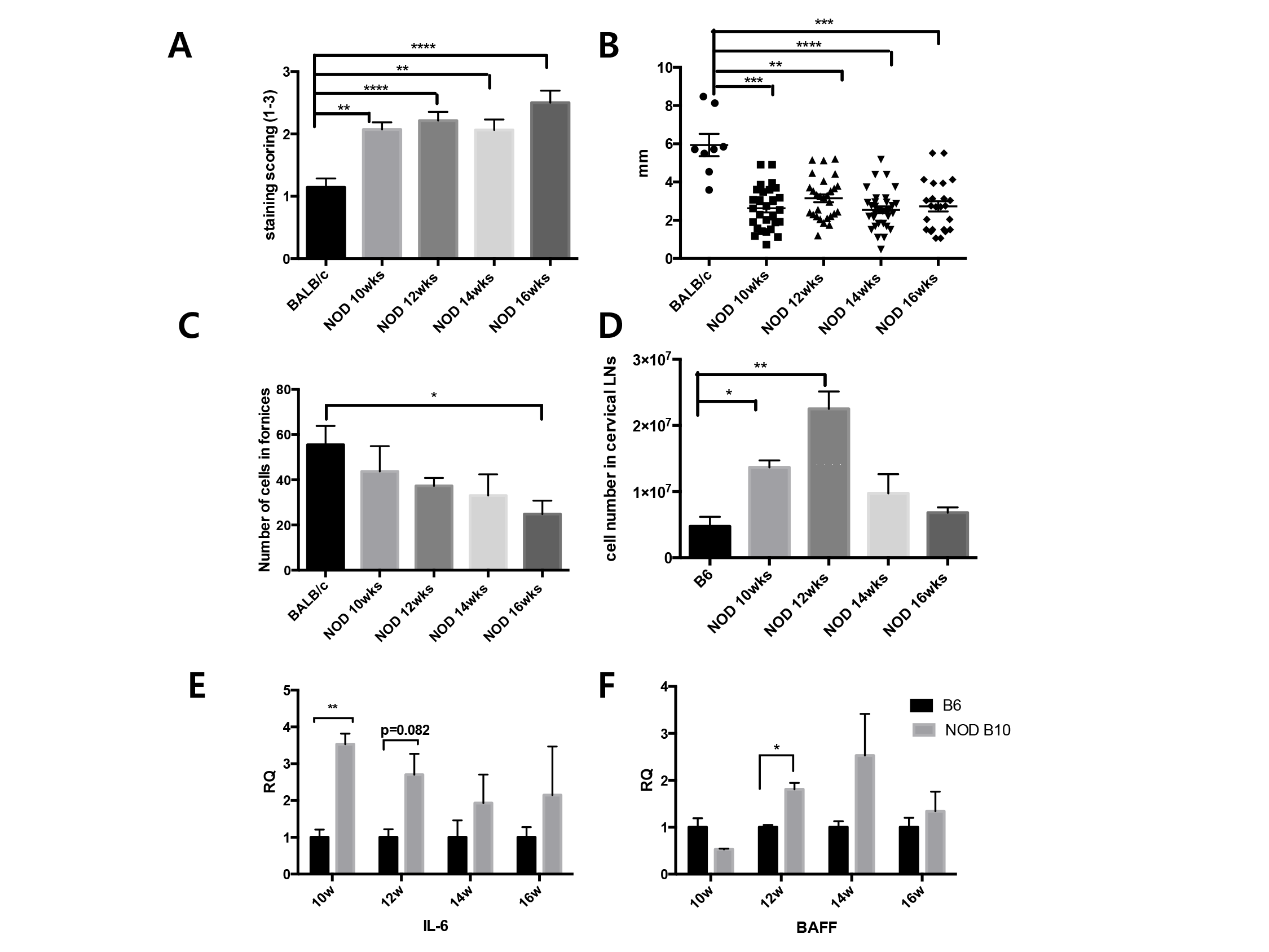

Supplement: S1 Fig — Clinical manifestation of dry eye and inflammatory responses were evident by 10 weeks. (A) Significantly decreased ocular staining score in NOD.B10.H2b mice at 10 weeks compared to BALB/c mice (Kruskal-Wallis test, **p < 0.01, ****p < 0.0001). (B) Phenol red thread test showing a significant decrease in tear secretion in NOD.B10.H2b mice at 10 weeks compared to BALB/c mice (Kruskal-Wallis test, **p < 0.01, ***p < 0.001, ****p < 0.0001). (C) Goblet cell density in conjunctiva of NOD.B10.H2b mice showing a decrease at 16 weeks compared to BALB/c mice (Kruskal-Wallis test, * p < 0.05). (D) Increased cell numbers in cervical lymph nodes of NOD.B10.H2b mice at 10 to 14 weeks compared to 10 week-old B6 mice (Kruskal-Wallis test, * p < 0.05, **p < 0.01). (E-F) Increased IL-6 and BAFF levels in NOD.B10.H2b mice at 10–12 weeks compared to controls (one-way ANOVA, *p < 0.05, **p < 0.01). RQ indicates a ratio of mRNA levels relative to controls. NOD, NOD.B10.H2b mice. (TIF) [file pone.0183678.s001.tif]

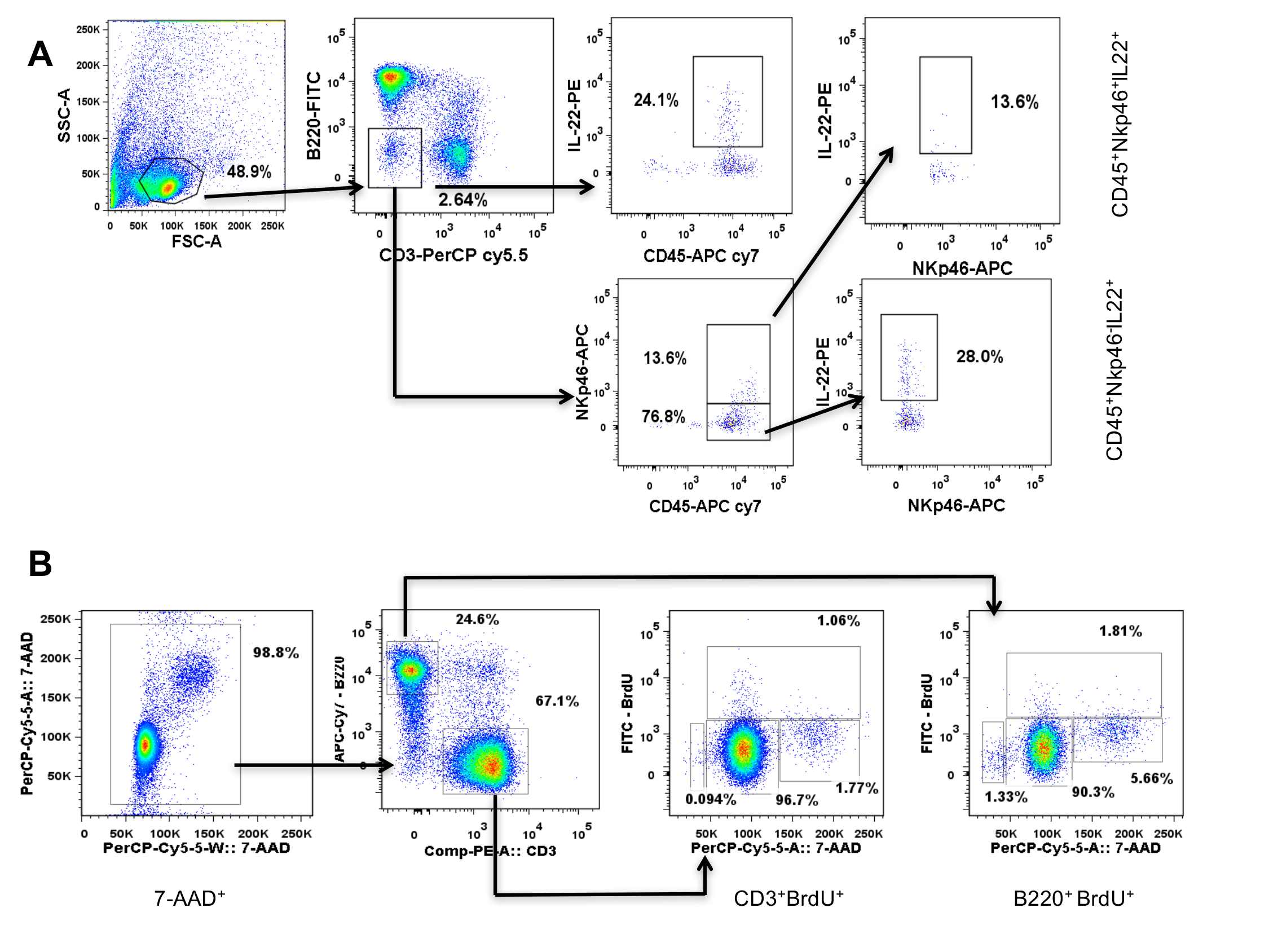

Supplement: S2 Fig — (A) ILC3s were negatively gated for anti-CD3 and anti-B220 antibodies and positively gated for anti-CD45 and anti-IL-22 antibodies. Subpopulation of NCR+ ILC3s (CD3-B220-CD45+Nkp46+IL-22hi cells) and NCR- ILC3s (CD3-B220-CD45+Nkp46-IL-22hi cells) were subsequently gated. (B) During the acquisition preview, gates were adjusted in the FSC-A vs. SSC-A plot, and the DNA 7-AAD-A voltage was adjusted to place the mean of the singlet peak (G0/G1) at 50,000 on the histogram. In addition, cell cycle gates were adjusted as needed to encompass the G0/G1, S, and G2/M populations. NCR, natural cytotoxicity receptor. (TIF) [file pone.0183678.s002.tif]

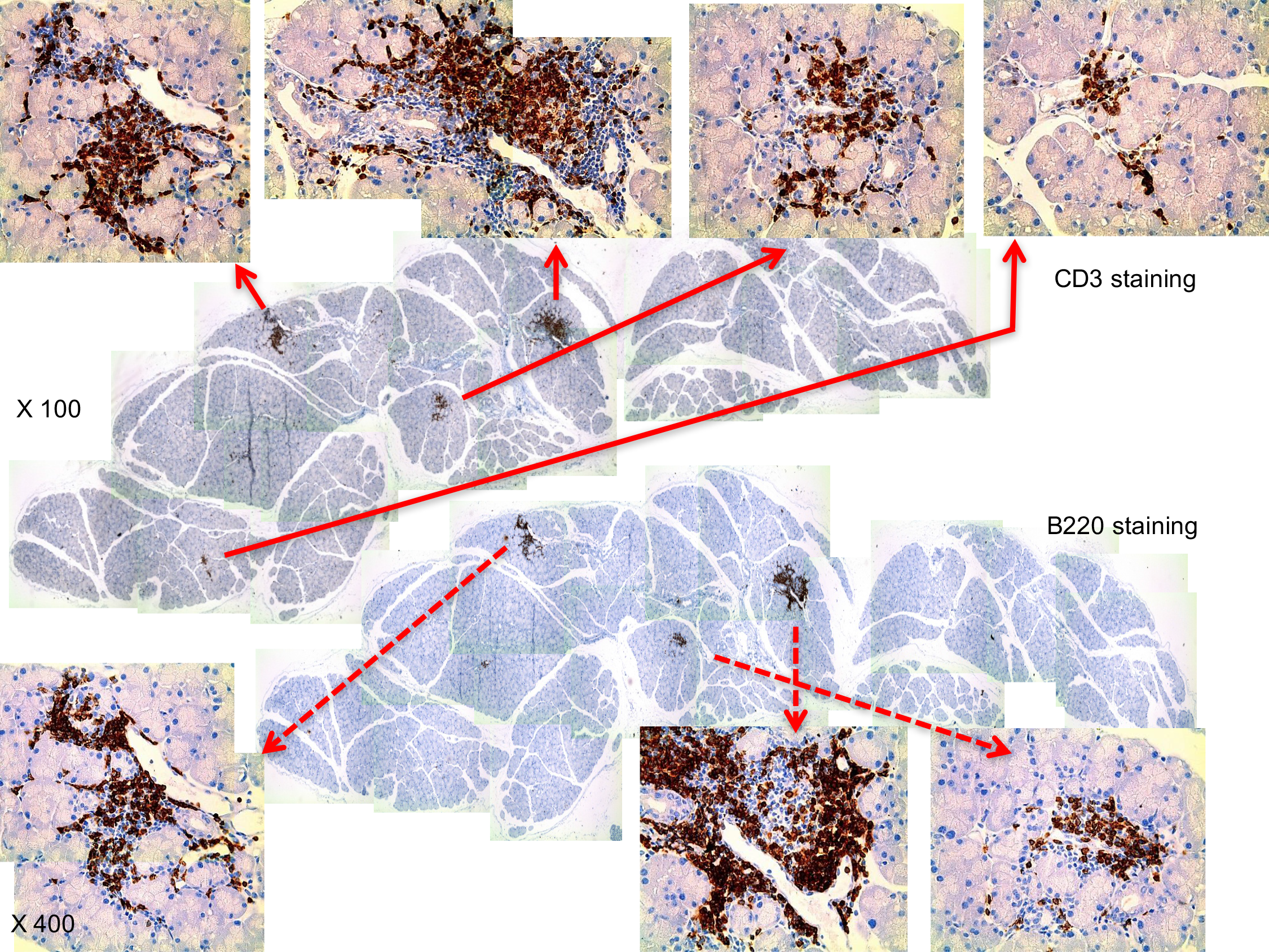

Supplement: S3 Fig — The infiltrating focus of CD3+ T cells (red arrows, upper panel, x400) almost matched that of B220+ B cells (red dashed arrows, lower panel, x400). (TIF) [file pone.0183678.s003.tif]
